# Synchronous Cardiac Fibroma and Medulloblastoma in Gorlin Syndrome: A Paradigmatic Case and Narrative Review

**DOI:** 10.3390/children12101314

**Published:** 2025-09-30

**Authors:** Marta Molteni, Gianluca Trocchio, Antonio Verrico, Maria Derchi, Nicola Stagnaro, Angela Di Giannatale, Paola Ghiorzo, Alessia Montaguti, Antonia Ramaglia, Claudia Milanaccio, Gianluca Piccolo, Maria Luisa Garrè

**Affiliations:** 1 Department of Neurosciences, Rehabilitation, Ophthalmology, Genetics, Maternal and Child Health, Università Degli Studi di Genova, 16126 Genoa, Italy; 2 Cardiology Unit, IRCCS Istituto Giannina Gaslini, 16147 Genoa, Italy; 3 Neuro-Oncology Unit, IRCCS Istituto Giannina Gaslini, 16147 Genoa, Italyclaudiamilanaccio@gaslini.org (C.M.); mluisagarre@gaslini.org (M.L.G.); 4 Radiology Unit, IRCCS Istituto Giannina Gaslini, 16147 Genoa, Italy; 5 Paediatric Haematology/Oncology Department, IRCCS Bambino Gesù Children’s Hospital, 00165 Rome, Italy; 6 Genetics of Rare Cancers, IRCCS Ospedale Policlinico San Martino, 16132 Genoa, Italy; 7 Emergency Department, Division of Neonatal and Pediatric Critical and Intermediate Care, IRCCS Istituto Giannina Gaslini, 16147 Genoa, Italy; 8 Neuroradiology Unit, IRCCS Istituto Giannina Gaslini, 16147 Genoa, Italy

**Keywords:** gorlin syndrome, cardiac fibroma, cardiac surgery, arrhythmia, medulloblastoma, *PTCH1*, narrative review

## Abstract

**Highlights:**

**What are the main findings?**

- In children with Gorlin syndrome, medulloblastoma (MB) and cardiac fibroma (CF) can occur synchronously.
- Timely initiation of MB therapy combined with conservative CF management can prevent delays in oncologic treatment while maintaining cardiac safety.

**What is the implication of the main finding?**

- A possible alternative to cardiac tumor excision can be considered, preferring a conservative approach and prioritizing MB treatment.
- Routine echocardiography should be performed in early-onset SHH-MB cases to detect CF early.

**Abstract:**

Background: Gorlin syndrome (GS) is a rare autosomal dominant disorder, associated with pathogenic PTCH1 or SUFU variants, predisposing to tumors such as basal cell carcinoma, medulloblastoma (MB), odontogenic keratocyst, and, rarely, cardiac fibroma (CF). MB occurs in ~5% of GS cases, typically in early childhood, while CF appears in 1–3%. Their coexistence in childhood is extremely rare. This report describes a pediatric GS case with synchronous MB and CF, focusing on the management priorities between oncologic and cardiac interventions. Methods: A 15-year follow-up is reported for a girl diagnosed at 22 months with desmoplastic/nodular MB and left ventricular CF. GS diagnosis was based on clinical features, imaging, and confirmation of a pathogenic PTCH1 variant (c.3306+1G>T). A literature narrative review on CF in GS was also conducted. Results: MB gross total resection was followed by chemotherapy, during which ventricular tachycardia episodes occurred, managed with cardioversion and antiarrhythmics. Given the favorable prognosis of early-treated MB in GS, oncologic therapy was prioritized. Cardiac status was monitored with ECG, Holter, echocardiography, and cardiac MRI. An adapted AIEOP protocol minimized cardiotoxicity. CF was managed conservatively, with no further arrhythmias and preserved ventricular function throughout 15 years. MB has not recurred. Conclusions: In GS patients with concurrent MB and CF, prioritizing MB treatment and adopting a conservative, closely monitored approach to CF can yield excellent long-term outcomes. In children with MB, especially syndromic forms, routine echocardiography is recommended to detect CF. This case underscores the value of multidisciplinary care in managing complex GS presentations.

## 1. Introduction

Gorlin syndrome (GS) is a rare autosomal dominant cancer predisposition syndrome, with prevalence estimated to be approximately 1/30,000–1/80,000, and is inherited in ~70–80% of cases [1]. Pathogenic variants most commonly affect *PTCH1*, less frequently *SUFU*, and rarely other Sonic Hedgehog (SHH) signaling pathway components (e.g., *PTCH2*). Germline or de novo variants usually manifest as loss-of-function or gain-of-function alterations that lead to aberrant cellular proliferation and tumor predisposition [2]. In normal physiology, SHH activity is tightly controlled by receptor proteins such as PTCH1, which inhibit the signaling activator SMO in the absence of ligand binding. In GS, germline variants in PTCH1, SUFU, or other SHH pathway genes result in constitutive activation of downstream effectors (notably the GLI transcription factors), promoting uncontrolled proliferation and survival of susceptible cell populations, thereby predisposing to multiple neoplasms [3]. In particular, malignant tumors associated with GS include basal cell carcinoma (BCC), a skin malignancy that can manifest prior to 20 years of age, and medulloblastoma (MB), generally with onset in the first years of life and typically with desmoplastic (DMB) or extended nodularity (MBEN) histology; benign tumors are odontogenic keratocyst of the jaw (OK), ovarian fibroma, and cardiac fibroma (CF). Thirteen diagnostic criteria are divided into six major (BCC, MB, OK, palmar/plantar pitting, lamellar calcification of the falx cerebri, a first-degree relative affected) and seven minor criteria (rib anomalies, polydactyly or other skeletal malformations, macrocephaly, cleft lip/palate, ovarian/cardiac fibroma, lymphomesenteric cyst, ocular abnormalities) [4,5,6,7].

Approximately 5% of all individuals with GS develop MB, with a peak of incidence between 1 and 2 years of age, thus differing from the sporadic form with onset mainly around the age of 7 years [8]. The tumor in GS tends to have a favorable prognosis [9]. GS predisposes patients to SHH-activated MB. In particular, PTCH1 germline variants (most common in GS) confer a moderate risk of developing SHH-MB, typically in childhood while SUFU variants (less common but high risk) are strongly predisposed to infant-onset SHH-MB [8]. Of note, MB was considered a minor criterion until 2011, when a consensus meeting changed it to major criteria and also allowed diagnosis with two minor criteria plus a major one [10]. Importantly, MB seems to be more frequent in patients with germinal pathogenic *SUFU* variants than *PTCH1* [6,11,12]. Consequently, according to the recommendations for cancer surveillance in GS, the screening of MB differs depending on molecular characterization: children with *SUFU* variants should undergo brain magnetic resonance (MRI) every 3–4 months during the first 3 years and then every six months until age 5 years; on the other hand, for those carrying a mutation in *PTCH1*, a periodic neurological examination is considered sufficient, with brain MRI to be performed only if neurological signs or symptoms appear [13]. Interestingly, in a large series of MB, pathogenic germline variants in *SUFU* or *PTCH1* were exclusively detected in group 2 MB (SHH) [14]; therefore, it is recommended that all children with a molecular diagnosis of SHH-MB should perform a genetic analysis of these two genes, especially when diagnosed before the age of 5 [13].

Cardiac fibroma is a rare but pathognomonic feature of GS, usually detected in the first years of life, when not already in utero, but diagnoses up to 60 years have been anecdotally reported [15,16]. It has been described in 1–3% of GS patients [5,17,18,19,20], and it may involve any structure of the heart, but it is mainly seen within the ventricles, occupying the chamber cavity and interdigitating with ventricular muscles; thus, it can replace the functional muscle mass, extending to the conduction system and therefore causing an arrhythmia [21]. Histology involves spindle cells with variable amounts of collagenized stroma and occasional foci of calcification, with frequent vascularization and few mitoses [22]. These intramural bulky tumors occur most commonly in the interventricular septum or ventricular free wall [23] and often contain calcifications at computed tomography (CT), due to scarce blood supply in the innermost part [24]. Central calcification is a highly characteristic/highly specific feature that distinguishes fibromas from rhabdomyomas [25]. At echocardiography, a large non-contractile and heterogeneous solid mass is usually detected [25], while cardiac MRI can show a hypo-isointense to myocardium mass on T1-weighted images and hypointense in T2-weighted images, with slow diffuse or heterogeneous contrast enhancement and a hypointense core [26]. Patients may clinically present with arrhythmias, heart failure, syncopal episodes, chest pain, cyanosis, and occasionally sudden death [27]. CF are benign solitary tumors, that do not progress to malignant counterparts but, differently from rhabdomyoma, do not regress spontaneously [28,29]. Therefore, surgical resection is usually suggested. No medical therapy is proven effective, except for different combinations of cardiological therapies, which are reported to have only partial efficacy in treating or preventing arrhythmias [30,31,32,33,34].

The main aim of this report is to present the conservative approach used in an ultra-rare case of concomitant MB and CF.

The subsequent narrative review synthesizes current evidence on the management of CF in the pediatric population. The relevant literature was identified through a comprehensive search of PubMed and Scopus up to June 2024, using keywords including “cardiac fibroma,” “children,” “pediatric,” “management,” “surgery,” and “outcomes.” Both clinical studies and case reports were included due to the rarity of the condition, while non-English articles without accessible translations were excluded. A narrative rather than a systematic review was chosen because of the rarity of the condition and the heterogeneous literature available.

## 2. Case Report

We report the case of a 17-year-old girl, admitted to the emergency department of our Hospital at the age of 22 months because of macrocrania and recurrent vomiting.

Second child of healthy, non-related parents of Russian origins, she was born at the gestational age of 39 weeks by cesarean section, due to macrocephaly. Polyhydramnios occurred during pregnancy.

At birth, the APGAR score was 10 at both 1′ and 5′. Growth parameters recorded on the first day of life were suggestive of macrocephaly (OFC 40 cm > 97th percentile), and weight was 4270 g (>97th percentile, LGA—large for gestational age). Family history revealed macrocephaly with frontal bossing in both her father and grandmother. Brain ultrasound examinations performed in the first year of life were unremarkable except for mild enlargement of the lateral ventricles.

**Figure 1:**
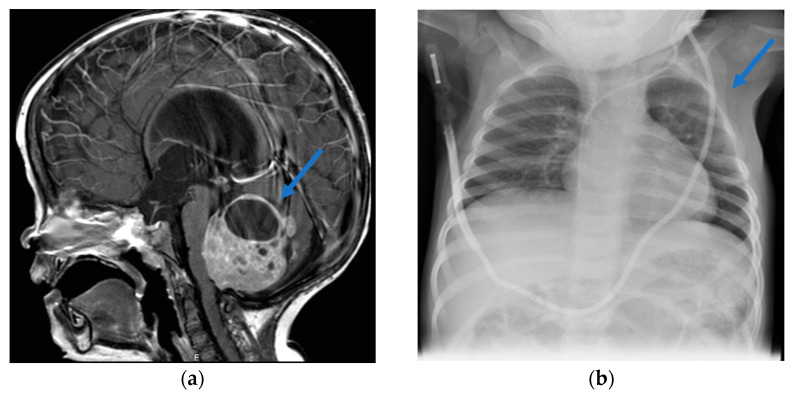
Clinical features of the patient at admission to our Institution. (**a**) Sagittal T1-weighted 3D Turbo Field Echo (TFE) contrast enhanced brain Magnetic Resonance Imaging (MRI) shows an expansive lesion involving the IV ventricle and the cerebellar vermis (blue arrow) and causing hydrocephalus. (**b**) Chest X-ray, showing a bifid rib (blue arrow) with an incomplete fusion of the III and IV left ribs.

At her admission to our department, the OFC was 57 cm (>97th percentile). She presented prominent frontal bossing, a plain hemangioma of the glabella, low nasal bridge, small nose with anteverted nares, outdistanced and sharp teeth, and clinodactyly of the second fingers of the feet. Neurological examination showed global hypotonia, more pronounced in the lower limbs, and psychomotor delay—walking only with support and with ataxic gait—with a developmental quotient of about 10 months. Brain and spinal cord MRI showed hydrocephalus and an expansive lesion involving the IV ventricle and the cerebellar vermis, without leptomeningeal metastases (Figure 1a).

Preoperative routine cardiac examination with electrocardiography was normal, showing only doubtful isodifasic T-waves in leads V4R and V1; thus, a control after the neurosurgical operation was suggested. External ventricular drainage was placed and gross total resection (GTR) performed. Histopathological examination was consistent with a desmoplastic/nodular medulloblastoma variant (WHO grade IV). Cerebrospinal fluid was unremarkable, and no tumor cells were detected at the cytological examination.

Considering the onset of medulloblastoma before 2 years of age, the histological pattern, the clinical features, and a family history positive for macrocrania, GS was suspected, and a genetic consult was requested. At chest X-rays, a bifid rib with an incomplete fusion of the III and IV left ones was noted (Figure 1b); ophthalmological, nephrological, and dermatological examinations were unremarkable. Karyotype resulted in normal (46 XX). Genetic analysis confirmed the diagnosis of GS, finding a pathogenic variant in *PTCH1* (c.3306+1G>T), a splice-site mutation in exon 19, absent in her mother. No genetic studies could be performed on her father, due to his refusal to perform analysis.

According to the Italian Association of Pediatric Haemato-Oncology (AIEOP) protocol for children under 3 years of age (cumulative doses: vincristine 2.9 mg/sqm, methotrexate 8 g/sqm, etoposide 2.4 g/sqm, cyclophosphamide 4 g/sqm, thiotepa 1.8 g/sqm), one month after surgery chemotherapy was started. The first course with methotrexate and vincristine was uneventful, but during hyperhydration preceding the second cycle (etoposide), ventricular tachycardia (VT) occurred (heart rate 240–260 bpm) without hemodynamic instability. Therefore, the chemotherapy plan was interrupted and a complete cardiac work-up was urgently performed: a 12-lead ECG displayed both a right branch block and a left anterior hemiblock; ventricular dissociation was detected with a trans-esophageal electrophysiologic study. Electrical cardioversion (2 J/Kg) stopped the VT, restoring sinus rhythm and showing flat T-waves in leads V4R and V1 and positive T-waves in V2.

**Figure 2:**
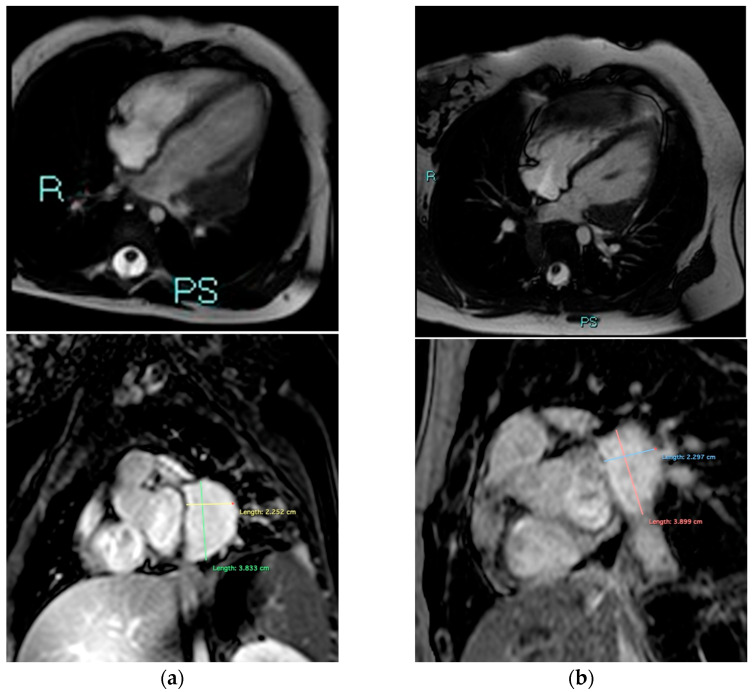
Cardiac MRI of the patient, performed at the onset of the cardiological symptoms (**a**) and after an 11-year-long follow-up (**b**), showing the stability of the size of the cardiac fibroma (main axis measures are shown in millimeters). (**Upper**) images: 4-chamber cine view, acquired with Steady-State Free Precession (SSFP) sequence. (**Lower**) images: Phase-Sensitive Inversion Recovery (PSIR) sequence on short axis plane, with a T1-weight (TI) of 320 ms (MRI scanner Philips 1.5T).

A Holter-ECG was performed, showing frequent ventricular ectopic polymorphous beats, but no episodes of ventricular or supraventricular tachycardia. An echocardiography revealed an echogenic lesion of the left ventricle free wall, 35 × 24 mm, neither exerting mass effect nor obstructing the ventricular outflow, with both normal coronaries and ventricular function. Computed tomography showed a mass in the free wall of the left ventricle without local calcifications. Cardiac MRI (CMR) confirmed a 25 × 43 × 38 mm mass, developing in the lower and middle portion of the left ventricle free wall, with eccentric development and intense homogeneous late enhancement (Figure 2a), indicative of cardiac fibroma (CF). In particular, before the intravenous (IV) contrast the mass was isointense with myocardium on T1-BB and slightly iso-hypointense on T2-BB. After IV contrast injection, it showed an intense and homogeneous enhancement and appeared to be encapsulated. Antiarrhythmic prophylaxis with propranolol was then started.

The case was discussed by a multidisciplinary team of cardiologists, cardiac surgeons, radiologists, and oncologists, who agreed on the following treatment strategy. Considering the optimal prognosis of desmoplastic MB in infants treated with chemotherapy, with overall survival of around 90%, priority was given to the pharmacological treatment of the brain mass, postponing any decision regarding a possible surgical approach to the cardiac tumor to the end of chemotherapy. In order to reduce cardiac toxicity, the chemotherapeutic regimen was modified, anticipating the drugs with minor cardiotoxicity while maintaining adequate dose intensity. Drug infusions were administered in the intensive care unit under continuous vital parameters and ECG monitoring. Three further VTs occurred during treatment; they were unresponsive to adenosine (up to 0.2 mg/kg), synchronized electrical cardioversion was successfully performed in all cases. Holter monitoring showed over 3000 polymorphic premature ventricular complexes (PVCs), including repetitive forms such as couplets and non-sustained ventricular tachycardia (NSVT), and propranolol was first replaced by and then added to amiodarone. ECG monitoring was conducted over several days to exclude QT prolongation, and thyroid function evaluated prior to treatment initiation and subsequently every six months. The lowest effective amiodarone maintenance dose was administered. A marked reduction in both the burden and complexity of ventricular ectopy was subsequently observed, with follow-up recordings showing approximately 100 isolated, predominantly monomorphic PVCs.

Of note, considering the severe fungal infection and gastrointestinal bleeding occurred during the first thiotepa cycle, the gross total resection performed upfront as well as the general good prognosis of DMB in GS, a multidisciplinary commission decided to withhold chemotherapy.

Lacking specific guidelines concerning CF in GS, a wait-and-see approach was chosen in agreement with cardiologists, with close tumor follow-up and regular cardiological assessment (ECG, stress-test, Holter monitoring, CMR). No further arrhythmias were recorded in a 15-year follow-up, and CMR confirmed CF stability (Figure 2b). Medulloblastoma has never recurred.

## 3. Discussion

Cardiac tumors in children are mainly benign (75%), with an incidence of 0.001% to 0.003%, representing the second most common histotype after rhabdomyoma [21,35]. The largest cohorts of pediatric patients affected by cardiac tumors date back 10 to 30 years ago and are mainly reported from the point of view of cardiac surgeons [36,37,38], including a small number or even no fibromas [39]. A uniform strategy to manage CF has not been established yet [40]. The main approach to cardiac tumors and, in particular, to CF remains the resection of the mass, but some cases are not good candidates due to the impossibility of preserving normal cardiac anatomy or function [41,42]. In the case of inoperable tumors, orthotopic heart transplantation can be an exceptional but adequate alternative and has increased its success rate in the last decade [37,43,44,45].

In most cases, the alleged reason for an operative approach is the malignancy of the tumor or, when considering CF, the high risk of lifelong malignant arrhythmias [31,46], even if data regarding arrhythmias in CF and their treatment are largely limited to case reports or small series [33,47,48,49]. Another reason to proceed to surgery can be the partial obstruction of the ventricular outflow [50], sometimes associated with valvular incompetence. Only two single case reports describe the spontaneous onset of VT in children (8 months and 6 years old) with GS and left ventricle CF [50,51]. As for adult patients, another case report refers to the possibility that presenting symptoms of CF in adults could be misinterpreted as an acute coronary syndrome [52]. Importantly, the risks directly related to cardiac surgery in children are unclear, with some authors reporting no fatalities among patients who underwent resection of benign masses [38] and others reporting a mortality rate of around 5%, mainly in cases of massive fibromas and due to heart failure [45]. The most common surgical complication in the case of massive tumors is related to mitral regurgitation [38]. A recent multicenter study by the European Heart Surgeon Association confirmed that surgical resection is indicated if the patient is symptomatic or when hemodynamic impairment occurs [42].

Burke et al. reported the clinicopathological findings in 23 patients affected by CF, with ages ranging from 1 day to 56 years, but only one of them was actually affected by GS; nineteen underwent surgery (considering both enucleation and biopsy), of whom six died (two cases during surgery, four after biopsy because of inoperable masses) [36]. When considering the only patient with GS, due to the extent of the tumor, cardiac transplantation was performed, with the patient being alive at a two-year follow-up.

In a retrospective single-center review, clinically significant arrhythmias in 173 pediatric patients with cardiac tumors were analyzed [31]. In total, 25 of them had CF, whose most common presenting symptoms were arrhythmias (32%), murmur, and abnormal chest X-ray (20% each). Of note, children with CF had the highest rate of arrhythmias among all tumor types, and T-wave abnormalities were commonly detected at ECG. There were 16 cases of VT (64% versus 6% in rhabdomyomas), with larger fibromas carrying a higher risk. A total of 50% of the VTs were intermittent episodes, but the other half required urgent intervention because of hemodynamic instability. In our case, arrhythmias occurred only during the initial, stressful phase of treatment, which included overhydration and polypharmacotherapy. Although ventricular and potentially life-threatening, they were hemodynamically well tolerated and controlled with antiarrhythmic medications. As concerns pharmacological therapy, in this same retrospective single-center review the efficacy of amiodarone was partial and limited to non-sustained VTs, while lidocaine seemed effective only when used in combination with procainamide. Two episodes of ventricular fibrillation were reported, both resolving after electrical cardioversion. CF was removed in 52% of cases, with no surgical death and no VT recurrence. The authors conclude that surgical excision for VT due to CF in selected patients is an effective management strategy [31]. In a similar cohort of 20 patients with CF, 75% presented with ventricular tachycardia with a median age at presentation of 4 years (range 5 months to 12 years); notably, 13 of them were also included in the study by Miyake previously reported [40].

As concerns amiodarone use in children, approximately 75% of patients may experience adverse effects with large doses, such as pulmonary and hepatic toxicities, hypo- or hyperthyroidism, hypotension (in case of rapid I.V. administration), bradycardia and/or heart block, hypotension, and QT prolongation. The lowest effective maintenance dose should be administered [53]. In cases of prolonged treatment, thyroid function should be assessed every six months, or earlier in patients at risk for, or with a history of, thyroid dysfunction [54]. As regards the genetic characterization of CF, little is known. Scanlan et al. described the cytogenetic (karyotype) and/or molecular findings (FISH) of three sporadic CF, none of them occurring in GS, but all carrying a loss of the chromosome 9 segments containing PTCH1 locus [55]. More recently, quantitative PCR was used to confirm a PTCH1 mosaic deletion in a CF diagnosed in a 4-year-old girl admitted to the hospital for tachyarrhythmia [56]. No studies are available in the literature about somatic variants of CFs in GS.

Importantly, in GS, genetic validation allows targeted management by linking genotype to phenotype: SUFU mutation carriers benefit from early and intensive neuroimaging surveillance due to high medulloblastoma risk, whereas PTCH1 mutation carriers require long-term dermatologic monitoring for basal cell carcinoma [6]. PTCH1-related GS is generally considered to have high penetrance, but clinical expressivity is highly variable. In our case, the presence of macrocephaly in the father and grandmother may reflect mild expressivity, even in the absence of more classic cutaneous or skeletal stigmata. Mosaicism could explain the limited phenotype and would have implications for recurrence risk in offspring. Therefore, clinical surveillance (dermatologic, odontogenic, and radiologic) should be recommended to the father, regardless of test status.

When a diagnosis of GS is plausible, ECG may help as the first screening for CF, showing right ventricular hypertrophy (although CF rarely occurs in the left ventricle, as for our patient) or intraventricular conduction defects [57]. Combining echocardiography and cardiac MRI with T1 and T2-weighted images (showing late enhancement), the diagnosis can be performed with relative certainty without performing a biopsy [36,58].

MB in GS is a rare entity, even rarer when associated with CF, with only one case described by Alanazi et al. [57]. Both our case and the one by Alanazi presented onset before 2 years of age, with DMB undergoing gross total resection, and a conservatively managed CF. Nevertheless, in the latter CF was asymptomatic (detected through surveillance) with an uncomplicated course not affecting MB treatment.

The concurrence of CF and MB causes an evident problem of which of the two should be treated first. On one side, the brain tumor needs fast and targeted therapy to reach both a good prognosis and outcome; on the other hand, CF is usually reported as a cause of serious, recurrent, and sometimes life-threatening arrhythmias.

It is peculiar that most MBs described in children with GS are DMB or MBEN; in a large monocentric study involving 82 cases of MB in patients aged less than 14 years, 27% were DMB and MBEN, and 52% of these occurred before the age of 3 years. MBEN was associated with GS in 5 of 12 cases and had a good prognosis. Desmoplastic histology and a more intensive treatment represented independent favorable prognostic factors in multivariate analysis [59].

Being CF non-regressing but the risk of arrhythmia variable, if the surgery risk/benefit ratio is not clear or the patient is paucisymptomatic, a conservative approach under a strict cardiological follow-up could be advised. Given the management implications of CF, including a delayed start of the chemotherapy, prompt identification of affected individuals is critical. Therefore, in young children with syndromic medulloblastoma, a routine echocardiogram should always be performed to rule out CF. At the same time, pediatric patients with CF should be carefully assessed for a diagnosis of GS, and brain MRI should be considered.

Over ten years, our patient was assessed by several cardiologists and cardiac surgeons. Surgery was not advised due to high procedural risk, absence of intracardiac flow abnormalities, no mass progression, and no recurrent arrhythmias. VT episodes occurred only with identifiable triggers, such as chemotherapy-induced hyperhydration or procedures under general anesthesia. The patient and family were counseled on the need for any future anesthetic procedures to be performed in a monitored setting, with avoidance of adrenergic drugs and at least 24 h of postoperative ECG monitoring. She remains on beta-blocker therapy (amiodarone stopped after two years, later switched to nadolol for its longer half-life). More recently, in view of her history and the need for improved long-term rhythm monitoring, implantation of a subcutaneous loop recorder was recommended.

## 4. Conclusions

In GS both CF and MB can occur at a very early age, but seldom together. Current literature is mainly in favor of cardiac surgery to avoid the risk of ventricular arrhythmias. At the same time, in case of MB, prompt treatment of medulloblastoma should take priority, while CF may be safely managed conservatively with close follow-up. The rarity of patients with these conditions makes it hard to gather robust evidence, carefully balancing scientific rigor with clinical practicality. Advanced methodologies, collaborations, and patient-centered approaches can be key to adopting adequate approaches in rare diseases.

Our case illustrates how treatment strategies can be tailored, balancing oncological outcomes with cardiac safety.

Children under 3 years with DMB should be evaluated for GS, and genetic testing extended to parents.

## Data Availability

The original contributions presented in the study are included in the article, further inquiries can be directed to the corresponding author.
